# SARS-CoV-2 seroprevalence among learners in grades 1–7, their parents and teachers in KwaZulu-Natal, South Africa: a cross-sectional study

**DOI:** 10.3389/fpubh.2025.1548945

**Published:** 2025-05-02

**Authors:** Reshmi Dassaye, Terusha Chetty, Brodie Daniels, Trisha Ramraj, Zakir Gaffoor, Elizabeth Spooner, Ncengani Mthethwa, Duduzile Faith Nsibande, Vuyolwethu Magasana, Khanya Mohlabi, Isaac Singini, Nomonde Gwebushe, Kubashni Woeber, Ameena Goga

**Affiliations:** ^1^HIV and Other Infectious Diseases Research Unit, South African Medical Research Council, Tygerberg, South Africa; ^2^Discipline of Public Health Medicine, University of KwaZulu-Natal, Durban, South Africa; ^3^Biostatistics Research Unit, South African Medical Research Council, Tygerberg, South Africa; ^4^Grants, Innovation and Product Development Unit, SAMRC, Tygerberg, South Africa; ^5^Department of Paediatrics and Child Health, University of Pretoria, Pretoria, South Africa

**Keywords:** COVID-19, SARS-CoV-2, learners, seroprevalence, long COVID

## Abstract

**Introduction:**

There is limited information on SARS-CoV-2 seroprevalence among children and adolescents in LMIC school settings. We aimed to assess (1) the seroprevalence of SARS-CoV-2 antibodies, (2) prevalence of self-reported or confirmed SARS-CoV-2 prior infections and, (3) COVID-19 symptoms (including long-COVID) among a cohort of primary school learners, their parents and teachers in a semi-rural school setting approximately 3-years into the COVID-19 pandemic.

**Methods:**

Learners in grades 1–7 attending two pre-selected schools in close proximity in the Ndwedwe area, iLembe district, KwaZulu-Natal, South Africa, their parents and teachers were invited to enroll into the COVID Kids Schools Study (CoKiDSS) – a cross-sectional survey conducted between May–August 2023. All participants provided informed consent, completed a questionnaire and provided a fingerprick of blood for SARS-CoV-2 antibody testing using the COVID-19 IgG/IgM Rapid Test. Statistical methods included descriptive analysis, jackknife-estimated seroprevalence and incidence (unadjusted and sensitivity-adjusted), and logistic regression using generalized linear models.

**Results:**

A total of 645 participants (i.e., 456 learners, 147 parents and 42 teachers) were enrolled into the survey. Overall SARS-CoV-2 IgG seroprevalence was 78% unadjusted to 81% adjusted with an increasing seropositivity trend, from learners to teachers (76% unadjusted to 79% adjusted in learners, 79% unadjusted to 82% adjusted in parents and 93% unadjusted to 97% adjusted in teachers). About 2.6% of learners tested IgM seropositive. Interestingly, 17% of the participants, including 20% learners, tested negative for SARS-CoV-2 antibodies. While only 16 participants (2.5% - 2 learners, 10 parents, and four teachers) self-reported a prior confirmed SARS-CoV-2 infection. Of these 2 learners (100%), eight parents (80%) and 4 teachers (100%) reported COVID-19 like symptoms that persisted for ≥28-days.

**Conclusion:**

We reported high SARS-CoV-2 IgG seroprevalence among learners in grades 1–7, their parents and teacher approximately 3 years into the COVID-19 pandemic which may be attributed to the snowball effect of multiple waves of infection in South Africa. However, only a small proportion of participants self-reported prior COVID-19 infection. This may be due to (1) recall bias and participants’ perception of low susceptibility to and severity of COVID-19, (2) limited access to SARS-CoV-2 testing, and/or (3) a high prevalence of asymptomatic infections.

## 1 Introduction

At the start of the coronavirus disease 2019 (COVID-19) South Africa (SA) like most countries swiftly implemented a comprehensive public health response including school closures from the 18th of March 2020, and a nationwide lockdown to curb the COVID-19 pandemic (1, 2). The government subsequently adopted a phased approach to reopening schools with a staggered grade return between June to August 2020, and a second round of school closures in 2021. The South African schooling system was officially re-opened (i.e., daily school attendance) on 2nd August 2021 (3). During this period, there were substantial losses in face-to-face teaching time and opportunities for remote learning were exceedingly limited. In low-middle income (LMIC) settings such as SA, there is sparse data on the number of COVID-19 infections and seropositivity in school-going children.

Children and adolescents aged 0–19 years comprise 37% of the South African population (4). The most recent report from the National Institute of Communicable Diseases (NICD) SA as of 4 December 2021, indicated that individuals aged ≤19 years made up 14.8% of SARS-CoV-2 tests, 12.5% of laboratory-confirmed COVID-19 cases, 5.0% of all COVID-19-associated admissions and 0.7% of COVID-19 associated in-hospital deaths (5). Preliminary evidence suggested that children are less susceptible to fatal severe acute respiratory coronavirus 2 (SARS-CoV-2) infection relative to adults and are generally asymptomatic or manifest mild, transient symptoms of COVID-19 (6). These early studies used SARS-CoV-2 polymerase chain reaction (PCR) tests which tested for active COVID-19 infection (6). Later studies utilized SARS-CoV-2 antibody tests (suggestive of prior infection) and demonstrated that children have a larger role in SARS-CoV-2 infection and transmission (7). Further, the rapid transmission of the SARS-CoV-2 omicron variant (B.1.1.529) between late 2021 and 2022 led to increased hospitalization rates among those aged 0–19 years (8–10). In many countries, children and adolescents were not prioritized in the initial COVID-19 vaccination program due to their low risk profile, vaccine safety concerns and parental hesitancy (11, 12). South African children aged 5–11 years at risk of severe COVID-19 were eligible to receive two doses of a COVID-19 specific vaccine as of 27 February 2023 (13). There is no available data on COVID-19 vaccination uptake among 5–11 year old South African children. However, UNICEF reported that as of 22 June 2022, only 30% of adolescents aged 12–17 years had received a COVID-19 vaccination in SA (14). Hence, children and adolescents remain the largest unvaccinated population in SA and globally.

South Africa like many LMICs has a paucity of data on SARS-CoV-2 seroprevalence among children and adolescents. Thus far, three SARS-CoV-2 seroprevalence community studies were implemented in SA during the beta, delta and start of the omicron waves, respectively (15–17). The overall findings indicate that the seroprevalence increased with each successive wave and that seroprevalence among adolescents and adults was comparable. Contrarily, younger children demonstrated a lower seroprevalence. This cross-sectional survey assessed SARS-CoV-2 seroprevalence among a triad of primary school learners, their parents/guardians (hereon referred to as “parents”), and teachers in a school setting approximately 3 years into the COVID-19 pandemic and 21–24 months post SA schools officially reopening. During this time, the SARS-CoV-2 omicron variant and its recombinants were in circulation.

## 2 Materials and methods

### 2.1 Study design, setting, and population

The protocol of this cross-sectional survey has been published (18). The cross-sectional survey is part of the COVID Kids School Study (CoKiDSS), a pilot study implemented between May 2023 and August 2023 in the Ndwedwe area, iLembe district, KwaZulu-Natal, SA. Ndwedwe is a semi-rural area with a population of about 165,826 (4). The target population for the cross-sectional survey were learners in grades 1–7 attending primary school, their parents and teachers. A convenience sample of two public primary schools that were large enough to contribute to the target sample size, in close proximity, and from the same catchment area was selected, to avoid heterogeneity, with the option of expanding, if needed. Based on prior seroprevalence data (17) indicating an approximate 40% seroprevalence, and assuming a 6% margin of error, 5% type 1 error rate, and design effect of 2.5, a sample of 640 participants was planned. The 640 were intended to be distributed as follows: 451 learners, 147 teachers and 42 parents.

Participation was voluntary and written informed consent was sought from teachers and parents for their own / their child’s participation, prior to enrollment. Assent was obtained from learners aged 8- < 18 years, and participation was contingent on obtaining both parental consent and assent. Only parental consent was required for learners 7 years of age. Participants received a voucher valued at about US $16 in accordance with local Ethics guidance, to cover the cost of time, inconvenience and participant expenses incurred.

### 2.2 Study procedures and data collection

Within the two pre-selected schools, all learners in grades 1–7, their parents and teachers were invited to participate in the survey. Study information was shared in the local vernacular with the school principal, teachers, parents and learners. Recruitment started in school 1 and then expanded to school 2. Enrolled learners, their parents and teachers completed an interviewer- or self-administered questionnaire that was captured using the Research Electronic Data Capture (REDCap) database (19, 20) and a fingerprick blood sample was collected. If the learner was enrolled in the survey, the parent completed the questionnaire on the learner’s behalf. Each questionnaire took between 20 and 30 min to complete. Information was collected on sociodemographic and basic health; self-reported COVID-19 history; self-reported symptoms of long COVID (COVID-19-like symptoms for 28-days or longer) (21) and COVID-19 vaccination status of learners, parents and teachers as well as household structure and household exposures.

### 2.3 Specimen collection and serological testing

The primary outcome of this survey was the seroprevalence of SARS-CoV-2 antibodies in a triad of learners in grades 1–7, their parents and teachers. A trained field worker collected a fingerprick blood sample from all participants to assess SARS-CoV-2 antibody seroprevalence using a South African Health Products Regulatory Authority (SAHPRA) approved point-of-care (POC) COVID-19 antibody test. The COVID-19 immunoglobulin (Ig)G/IgM Rapid Test Cassette from Orient Gene Biotech, a lateral flow immunochromatographic assay was utilized - as per the manufacturer’s package insert, the test had a relative sensitivity of 96.2% and a relative specificity of 100% (22). The test was performed with 10 μL of whole blood. The samples were deposited in the specimen well of the test device and the sample buffer was added to the buffer well. Air bubbles were avoided. The results were read in 10 min. IgG and IgM were represented by two separate bands and were read visually.

### 2.4 Statistical analysis

A descriptive analysis of school and participant characteristics was conducted using proportions for categorical variables. For normally-distributed data we report means with standard deviations and for skewed data we report medians with interquartile range (IQR). We estimated both unadjusted and adjusted total seroprevalence and cumulative incidence, stratified by age and gender. Adjusted estimates accounted for the sensitivity of the POC test using the following formula:

True Prevalence=Observed Prevalence/Sensitivity

Seroprevalence estimates, including prior and current COVID-19 symptoms, are reported with 95% confidence intervals (CIs), with standard errors computed using jackknife resampling methods. To assess changes in seroprevalence, symptoms, health, and socio-demographic factors within the cohort, we used generalized linear models with logistic regression to estimate the log odds of seropositivity (IgG or IgM positivity). The selection of covariates for multivariate adjustment was based on both statistical significance in univariate analyses (*p* < 0.20) and *a priori* selection informed by existing literature and biological plausibility. We report odds ratios (ORs) with 95% CIs and *p*-values. Model fit was assessed using goodness-of-fit statistics (Deviance and Likelihood Ratio Tests), to evaluate the appropriateness of the logistic regression models. Missing data were handled using a complete-case analysis approach, and sensitivity analyses were conducted to evaluate the impact of missingness on key estimates. All statistical analyses were performed using R (version 4.0.3) with the tidyverse suite and Stata 16 statistical analysis package.

## 3 Results

### 3.1 Characteristics of the participating schools

Two out of four pre-selected primary schools in the Ndwedwe area, iLembe district, KwaZulu-Natal, SA, agreed to participate in the CoKiDSS. Hereon the two schools’ will be called primary school 1 and primary school 2. Both primary schools offered classes in grades 1–7 (Table 1). Primary school 1 was a larger educational institution and was 3.5 times the size of primary school 2, as evident by the number of pupils per grade (Table 1). Recruitment started in school 1 and subsequently expanded to school 2; thus 80.9% of participants were recruited from primary school 1 whereas 19.3% of participants were recruited from primary school 2 (Table 1); school 1 and school 2 contributed 29.4 and 25.4% of their learners respectively, and 62.5 and 29.2% of their teachers, respectively. The schools were in close proximity and from the same catchment area to avoid heterogeneity; as per the study protocol, the analysis pooled participants across schools.

**Table 1:** Characteristics of the school, and enrolled population per school.

	School 1			School 2		
	*n*	%	Number and proportion enrolled from school 1 *n* (%)	*n*	%	Number and proportion enrolled from school 2 *n* (%)
Number of learners per school?	1,252	100	368(29.4)	351	100	89(25.4)
Number of ^*^teachers per school	56	100	35(62.5)	24	100	7(29.2)
Total number of learners and ^*^teachers enrolled per school			403 (30.8)			96 (25.6)
Number of learners per grade						
Grade 0	137	10.9	–			
Grade 1	162	12.9	48 (29.6)	39	10.5	–
Grade 2	152	12.1	56 (36.8)	34	9.2	9 (26.5)
Grade 3	171	13.7	52 (30.4)	38	10.3	6 (15.8)
Grade 4	122	9.7	45 (36.9)	56	15.1	12 (21.4)
Grade 5	161	12.9	44 (27.3)	66	17.8	22 (33.3)
Grade 6	193	15.4	72 (37.3)	31	8.7	8 (25.8)
Grade 7	154	12.3	51 (33.1)	51	13.8	23 (45.1)
Number of teachers per grade						
Grade 0	4	14.3	8 (200.0)	1	10.0	–
Grade 1	4	14.3	5 (125.0)	1	10.0	1 (100.0)
Grade 2	4	14.3	5 (125.0)	2	20.0	1 (100.0)
Grade 3	4	14.3	4 (200)	2	20.0	1 (50.0)
Grade 4	3	10.7	4 (133.3)	1	10.0	1 (50.0)
Grade 5	3	10.7	5 (166.7)	1	10.0	1 (100.0)
Grade 6	3	10.7	4 (133.3)	1	10.0	1 (100.0)
Grade 7	3	10.7	8 (200.0)	1	10.0	1 (100.0)
Number of learners and ^*^teachers enrolled per school per ^$^sample size target			403 (80.9%)			96 (19.3%)

^*^Teachers = teachers and teaching assistants. ^$^Sample size target refers to the sample size allocation for learners and teachers only (i.e., 456 learners and 42 teachers).

### 3.2 Characteristics of the study participants (i.e., learners, their parents and teachers)

**Figure 1:**
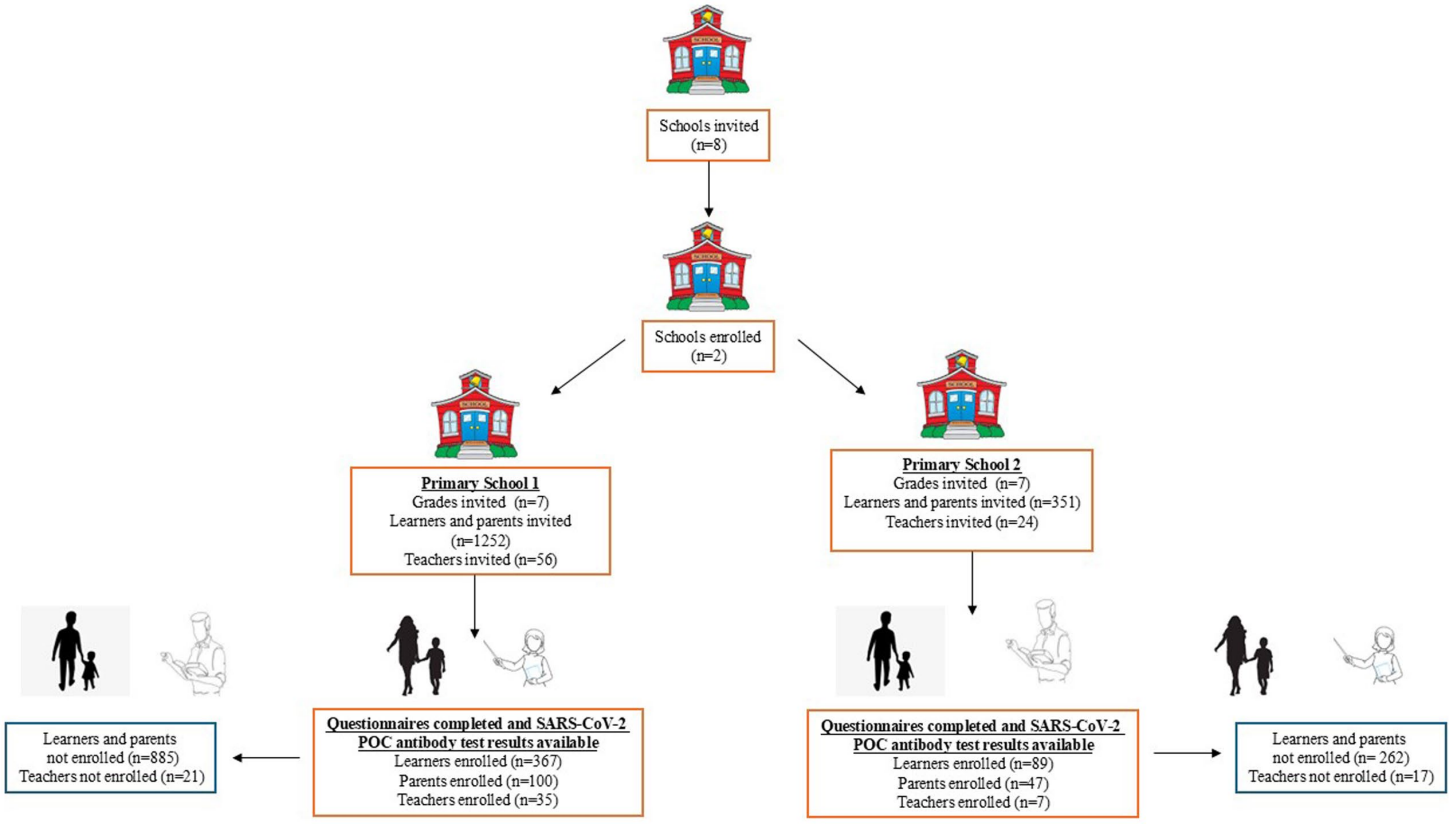
Flow chart of participants enrolled in the CoKiDSS - cross-sectional survey.

In total, 645 participants (456 learners, 147 parents and 42 teachers) from two pre-selected primary schools, were enrolled into the survey between May–August 2023 (Table 2). Figure 1 shows the flowchart of enrolled participants with available questionnaire information and serological test results. The participation among learners in senior (grades 4–7) primary was greater than junior (grades 1–3) primary (senior primary: 50.9% vs. junior primary: 40.1%). About half of learner- and parent- participants were female (learners *n* = 242, 53.1%, and parents *n* = 133, 90.0%), respectively, while approximately 83% of teachers were female (*n* = 35). The majority of learners were between 7 and 15 years of age (*n* = 407), 89.4%: 172 (38%) were 7–10 years of age and 235 (51.4%) learners were 11–15 years of age. Most parent participants were between 30 and 59 years of age (*n* = 109, 74.1%: 58 (39.5%) parents were 30–39 years of age and 51 (34.7%) parents were 40–59 years of age). Similarly, a greater proportion of teacher participants were between 40 and 59 years of age (*n* = 33, 78.6%) Over 99% of participants self-identified as Black African ethnicity (learners’: *n* = 452, 99.1%; parents: *n* = 146, 99.3%, and teachers *n* = 42, 100%). Less than half the parents completed secondary or university and 78% of parents were unemployed (*n* = 115, 78.2%). Participants self-reported being diagnosed with the following conditions: HIV: 7 (1.5%) learners, 36 (24.5%) parents and 5 (11.0%) teachers; current TB: 1 (0.2%) learner and 1 (0.7%) parent; previous TB: 2 (0.4%) learners and 7 (4.8%) parents; diabetes: 7 (4.8%) parents and 3 (7.1%) teachers and other conditions; 2 (0.4%) learners, 17 (11.6%) parents and 5 (11.0%) teachers. Additionally, participants self-reported taking the following concomitant medications: ARV/ART: 5 (1.1%) learners, 49 (33.3%) parents and 3 (7.1%) teachers; Bactrim: 1 (0.68%) parents and 1 (2.4%) teachers; TB medication: 1 (0.2) learners and other medications; 1 (0.25%) learner, 18 (12.2%) parents and 8 (19.0%) teachers. Only 2 (0.44%) learners, 10 (6.80%) parents and 4 (9.52%) teachers self-reported previously being infected with SARS-CoV-2 or self-reported previous COVID-19. Two learners (0.4%), 60 parents (41.0%) and 33 teachers (79.0%) reported having received a COVID-19 vaccination at the time of the cross-sectional survey (Table 2).

**Table 2:** Characteristics of the study participants by group.

Individual level characteristics	Learners in grades 1–7		Parents		Teachers	
	*n*	%	*n*	%	*n*	%
	456	100	147	100	42	100
Grade						
Junior primary (grades 1–3)	183	40.1				
Senior primary (grades 4–7)	273	50.9				
Gender (sex)						
Male						
Female	214	46.9	14	9.5	7	16.7
Other	242	53.1	133	90.5	35	83.3
Age						
< 7	46	10.1				
7–10	172	38.0				
11–15	235	51.4				
16–18	3	0.7				
18–29			29	19.7	8	19.1
30–39			58	39.5	15	35.7
40–59			51	34.7	18	42.9
≥60			9	6.1	1	2.4
Ethnicity (race)						
Black African	452	99.1	146	99.3	42	100
Indian	1	0.48	-	-		
Colored	2	0.48	-	-		
White	1	0.2	1	0.7		
Other	-					
Level of education						
No formal education			4	2.7		
Junior Primary School (grades 1–4)			13	8.8		
Senior Primary School (grades 5–7)			9	6.1		
Some secondary school (grades 8–12)			55	37.1		
Completed secondary school (grades 8–12)			59	40.2		
Some university/technical education			4	2.7		
Completed university/technical education			2	1.4		
National certificate/trade			1	0.7		
Employment status						
Employed, part-time			18	12.2		
Employed, full-time			13	8.8		
Unemployed			115	78.2		
Other			1	0.7		
Comorbidities						
HIV	7	1.5	36	24.5	5	11.9
TB (current)	1	0.2	1	0.7	0	0.0
TB (Prior)	2	0.4	7	4.8	0	0.0
Diabetes Mellitus	0	0.0	7	4.8	3	7.1
^*^Other comorbidities	2	0.41	16	10.9	4	9.5
Concomitant medications						
ARVs/ART	5	1.1	49	33.3	3	7.1
Bactrim	0	0.0	1	0.7	1	2.4
TB meds	1	0.2	0	0.0	0	0.0
^#^Other concomitant medications	1	0.2	14	10.0	6	14.3
Previous COVID-19 infection						
Yes	2	0.44	10	6.8	4	9.5
No	454	99.56	137	95.8	38	90.5
Vaccination status						
^$^Vaccinated	2	0.4	60	41.0	33	79.0
Unvaccinated	454	99.6	86	59.0	9	21.0

*Other comorbidities include chronic kidney disease, chronic liver disease, heart disease, hypertension, asthma, chronic lung disease, rheumatological disease and obesity. ^#^Other concomitant medication includes steroids, anti-inflammatories, antihypertensives, hormonal treatment, antibiotics, aspirin/warfarin/heparin and anticoagulants. ^$^Vaccinated refers to having received at least one dose of a COVID-19 vaccine.

### 3.3 Household level characteristics of the participants

Four hundred and fifty-seven participants provided household data, and the median number of people per household was 5 (2), the median number of rooms per household was 4 (3.0) and the median number of rooms used for sleeping per household was 3 (1.1) (Table 3). More than half the participants (269 (58.9%)) reported residing with 1–5 household members (Table 3). Sixty three participants (13.8%) reported having 1–2 rooms per household and 209 participants (45.8%) reported having 3–4 rooms per household (29.6%). About half of households reported using 1–2 rooms for sleeping - 254 participants (55.6%) and 181 participants (38.6%) reported using 3–4 rooms for sleeping.

**Table 3:** Summarizes the household level characteristics of the participants.

Household level characteristics	Overall *n*	%
# of household members		
1–5	269	58.9
6–10	183	40.0
≥11	5	1.1
# of rooms per household		
1–2	63	13.8
3–4	209	45.8
5–6	135	29.6
>6	50	11.0
# of rooms used for sleeping per household		
1–2	254	55.6
3–4	181	39.6
5–6	20	4.4
>6	2	0.4
Household level characteristics	Median (IQR)	
# of household members	5 (2.0)	
# of rooms per household	4 (3.0)	
# of rooms used for sleeping per household	3 (1.1)	

### 3.4 SARS-CoV-2 antibody seroprevalence

Among all participants, the overall seroprevalence of SARS-CoV-2 IgG antibodies was 78.0% -unadjusted and 81% - adjusted whereas IgM was 1.9% - unadjusted and 2.0 – adjusted and 16.5.% unadjusted and 17.7% - adjusted of participants tested negative (*p* < 0.001) (Table 4). The IgG seroprevalence was heterogeneous across all groups, ranging from 76% - unadjusted to 79% adjusted (*n* = 346) in learners, 79% - unadjusted to 82% adjusted (*n* = 116) in parents and 93% - unadjusted to 97% adjusted (*n* = 39) in teachers, whereas only 2.6% (*n* = 12) of learners tested IgM seropositive. Interestingly, 20% - unadjusted to 21% adjusted (*n* = 92) of learners, 8.2% - unadjusted to 8.5% adjusted (*n* = 12) of parents and 7.1% - unadjusted and 7.4% adjusted (*n* = 3) of teachers tested SARS-CoV-2 antibody negative (Table 4). No gender differences in IgG and IgM seroprevalence were observed among the learners, parents and teachers. Similarly, no age differences in IgG and IgM seroprevalence were observed among learners, their parents and teachers, respectively.

**Table 4:** Overall SARS-CoV-2 antibody seroprevalence and by group unadjusted and adjusted for POC sensitivity of 96.2%.

Group	Overall (*n* = 644^1^) Unadjusted (Adjusted)	Learners in grades 1–7 (*n* = 456^1^) Unadjusted (Adjusted)	Parents (*n*= 147^1^) Unadjusted (Adjusted)	Teachers (*n* = 42^1^) Unadjusted (Adjusted)	*p*-value^2^ Unadjusted (Adjusted)
Antibody result					<0.001 (<0.001)
Indeterminate	25 3.9% (4.1%)	6 1.3% (1.4%)	19 12.9 (13.5%)	0 0 (0.0%)	
IgG positive	501 77.7% (81.1%)	346 76% (79.0%)	116 78.9% (82.1%)	39 92.9% (96.7%)	
IgM positive	12 1.9% (2.0%)	12 2.6% (2.7%)	0 0 (0%)	0 0(0.0%)	
IgG and IgM Negative	107 16.5% (17.7%)	92 20.1% (20.8%)	12 8.2% (8.5%)	3 7.1% (7.4%)	

^1^*n* (%). ^2^Fisher’s exact test.

### 3.5 Long COVID

Among all participants, only two learners (0.4%) reported having had prior COVID-19 and both reported COVID-19-like symptoms for 28-days or longer; 10 (6.8%) parents had prior COVID-19 and eight experienced COVID-19-like symptoms for 28-days or longer while four teachers had prior COVID-19 and all four experienced symptoms longer than 28 days. Hence, 80–100% of participants that self-reported previous COVID-19 infection also self-reported COVID-19-like symptoms for 28-days or longer. There were no participants that reported to have missed school or work, respectively due to the prolonged COVID-19-like symptoms. All parents and teachers who reported long COVID-19-like symptoms (eight parents and four teachers reported earlier) had spent a median of 5 days (IQR = 4, 5) in hospital for treatment. Of the two learners that reported long COVID, one learner was SARS-CoV-2 IgG seropositive, and one was negative, six of eight parents tested negative, and all four teachers tested IgG seropositive at the time of the survey.

### 3.6 Factors associated with seropositivity

In the unadjusted analysis, four factors were identified as risk factors for SARS-CoV-2 antibody seropositivity (Table 5). Among these, females had a two-fold odds of being IgG/IgM positive compared to their male counterparts [Odds ratio: 2.3, 95% CI (1.42, 3.8), *p* < 0.001]. The group (pairwise) that one belongs to also had a statistically significant association with seropositivity as well as vaccination status [Odds ratio: 4.43, 95% CI (1.58, 18.5), *p* = 0.014] and age [Odds ratio: 1.04, 95% CI (1.02, 1.07), *p* < 0.001] (Table 5) in the univariate analysis. When adjusted, no statistically significant association for seropositivity was seen among these factors. This could be as a result of confounding variables.

**Table 5:** Risk or protective factors associated with IgG and IgM SARS-CoV-2 antibody seropositivity estimated using univariate and multivariate logistic regression.

Characteristic	Univariate odds ratios estimates			Multivariate odds ratios		
	OR^1^	95% CI^1^	*p*-value	OR^1^	95% CI^1^	*p*-value
Gender						
Male	–	–		–	–	
Female	2.32	1.43, 3.77	<0.001	0.97	0.36, 2.49	>0.9
Group: learner vs. parent						
Learner	–	–		–	–	
Parent	2.65	1.43, 5.33	0.003	2.54	0.37, 18.1	0.3
Group: learner vs. teacher						
Learner	–	–				
Teacher	2.65	1.43, 5.33	0.003			
Group: teacher vs. parent						
Teacher	–	–				
Parent	0.38	0.19, 0.70	0.003			
Vaccination status						
No	–	–		–	–	
Yes	4.43	1.58, 18.5	0.014	1.86	0.45, 9.76	0.4
Age	1.04	1.02, 1.07	<0.001	1.00	0.95, 1.06	>0.9
No. of people in the house						
1–4 people	–	–		–	–	
5 people	0.56	0.29, 1.10	0.084	0.53	0.16, 1.87	0.3
> 5 people	0.66	0.38, 1.12	0.13	0.40	0.14, 1.03	0.065
No. of rooms in the house						
1–3 rooms	–	–		–	–	
4 rooms	0.68	0.37, 1.27	0.2	0.50	0.18, 1.31	0.2
> 4 rooms	0.63	0.34, 1.19	0.2	0.93	0.30, 2.94	0.9
Self-reported HIV status						
Negative	–	–		–	–	
Positive	1.3	0.56, 3.48	0.6	0.64	0.18, 2.62	0.5

^1OR, Odds ratio; CI, Confidence interval.^

## 4 Discussion

### 4.1 Key findings

This cross-sectional survey aimed to assess SARS-CoV-2 seroprevalence in a triad of learners in grades 1–7, their parents and teachers, about 3 years post the start of the COVID-19 pandemic and ~21–24 months post the resumption of daily school attendance in SA. Both IgM and IgG antibodies were evaluated using a qualitative POC test, affording an appraisal of both recent and previous infection, respectively. The estimated half-life of IgM and IgG antibodies against SARS-CoV-2 range from 1 to 4 months and 6–18 months, respectively post infection (23). Overall, SARS-CoV-2 IgG seroprevalence was 78% (unadjusted) and 81% (adjusted), with an upward trend in seropositivity among learners (76% - unadjusted and 79% - adjusted), their parents (79% - unadjusted and 82% - adjusted) and teachers (93% - unadjusted and 97% - adjusted). We observed a higher seroprevalence of SARS-CoV-2 IgG among children compared to the last community serosurvey implemented in the Gauteng province of SA, from 22 October to 9 December, 2021with the omicron variant in circulation (76% - unadjusted and 79% - adjusted vs. 56.2%) (17). However, unlike Madhi et al. (17), the seroprevalence was comparable in children relative to adults in CoKiDSS (Madhi: 56.2% vs. 79.7% and CoKiDSS: 76% - unadjusted and 79% adjusted vs. 79% -unadjusted and 82% adjusted) due to the high transmissibility of the omicron variant (B1.1.529) and its recombinants (24). A small proportion of learners (2.6%) tested IgM positive during the duration of the cross-sectional survey. The overall high SARS-CoV-2 IgG seropositivity reported in this survey may be reflective of the cumulative effect of multiple waves of infection which may have occurred between January 2022 to August 2023 in parallel to the resumption of daily school attendance in SA. Teachers had the highest SAR-CoV-2 IgG seropositivity and this may be attributed to either a higher proportion of teachers being vaccinated against COVID-19, slower weaning of antibody response post exposure or due to ongoing transmission in this setting. A discordance in self-reported SARS-CoV-2 infection and seroprevalence was also observed (i.e., 2.5 vs. 78% - unadjusted and 81% adjusted). There may be several reasons for this finding including (1) recall bias influenced by social desirability and perceived perception of low susceptibility and severity of COVID-19; (2) poor access to SARS-CoV-2 testing and/or (3) a high proportion of asymptomatic infections. The latter cause is consistent with the PHIRST study, which reported a high rate of asymptomatic infections (85.3%) in individuals of all ages (25).

COVID-19 preventative measures and restrictions were relaxed in SA from April 2022, and we observed significant ongoing transmission during the 2023 study period. A small proportion of participants reported having had a prior COVID-19 infection (two learners, 10 parents, and four teachers). Among these, most experienced COVID-19-like symptoms that persisted for 28 days or longer: 2 out of 2 learners (100%), 8 out of 10 parents (80%), and 4 out of 4 teachers (100%). A meta-analysis and systematic review of long COVID children and adolescents showed that the pooled prevalence of any long COVID was 23.36% (95% CI 15.27–32.53) (26) whereas the meta-analysis and systematic review of long COVID in adults demonstrated that 45% of COVID-19 survivors, regardless of hospitalization status, were experiencing a range of unresolved symptoms at ∼ 4 months (27). During the duration of the survey, South African children aged 5–12 years of age were only eligible for a COVID-19 vaccination if they were at risk of severe COVID-19. Hence, only two learners reported having received a COVID-19 vaccination while more than 50% of parents and teachers were vaccinated. We also observed in the univariate analysis that age, gender, group (pairwise) and vaccination were potential risk factors for seropositivity. However, after adjusting for the means in the multivariate analysis, there were no significant differences due to confounding variables. Whereas another study demonstrated that potential risk factors for COVID-19 during the omicron variant included age > 65 years of age, male gender, and underlying medical conditions including hypertension and mental disorders compared with patients without these risks (28).

## 5 Strengths and limitations of the study

This study provides the first snapshot of SARS-CoV-2 seroprevalence among learners in grades 1–7 attending public primary schools in SA, their parents and teachers in a LMIC setting with a high HIV/TB prevalence. However, this study is not without limitations. Firstly, the study was conducted approximately 14–17 months post the termination of the National State of Disaster in SA when COVID-19 restrictions were lifted, and most preventative measures were relaxed, which could impact the outcome of the study. This was a pilot study and given the limited number of schools and geographic area, and the discrepancy between self-reported confirmed COVID-19 and antibody positivity the results may not be generalizable to all settings and provinces across South Africa. Little heterogeneity in the findings precluded modeling and in-depth multivariable analysis. This seroprevalence study measured past infection and we cannot ascertain when the infections occurred except for a broad time window, nor can we establish transmission dynamics within the school setting or transmission in the households. The finding of a substantial proportion of participants with confirmed prior SARS-CoV-2 infection reporting long COVID is based on a small sample size, which may limit the generalizability and robustness of the long-Covid-19 results.

We cannot exclude selection-bias due to increased willingness to participate due to the participant reimbursement. Reimbursement vouchers were issued to all participants to cover travel, inconvenience and expenses. Although most parent participants were unemployed, we have no reason to believe that there is a difference in seroprevalence between learners of employed versus unemployed participants. All learners interact and live in the same area, regardless of parental employment status. The SARS-CoV-2 pandemic demonstrated that the virus spreads across social, economic, and geographic boundaries (29). Thus, we believe that the antibody results are generalizable to other school populations globally, as SARS-CoV-2 circulation is not restricted by border or other controls, and the rapid antibody tests used in this study were not variant-specific. Lastly, we assessed SARS-CoV-2 seroprevalence using a COVID-19 point-of-care antibody test and presented qualitative results, further work may be required to assess SARS-CoV-2 seroprevalence using a quantitative test to further understand antibody protection and waning over time.

### 5.1 Implications for research and public health

This study contributes to the limited data on SARS-CoV-2 seroprevalence in LMIC school settings, offering insights into the pandemic’s evolution in this population and allowing for inferences about similar school populations in the region. Findings indicate that the majority of this semi-rural population have been exposed to SARS-CoV-2, exhibiting a persistent immunological response. The public health implications suggest that in semi-rural, low-resource settings, reported SARS-CoV-2 infections may underestimate actual virus exposure, with asymptomatic or undiagnosed cases being more prevalent than expected. Rapid antibody tests proved to be a feasible and well-accepted strategy among both children and adults, highlighting their potential utility in school settings for infection assessment and in future pandemics. Additionally, teachers represent a high-risk group, exhibiting higher antibody positivity than both parents and learners, reinforcing the rationale for their prioritization in COVID-19 vaccination strategies. Lastly, this study strengthened community engagement and local leadership in affected communities, fostering collaboration and supporting sero-surveillance efforts for preparedness and response to current and future pandemics.

## 6 Conclusion

The high SARS-CoV-2 IgG seropositivity reported in this paper provides evidence for the augmenting effect of multiple waves of SARS-CoV-2 infection despite the low proportion of self-reported prior COVID-19. Our data show that even in this semi-rural setting, 3 years into the COVID-19 pandemic and ~21–24 months after the official reopening of SA schools the majority of learners, parents and teachers were exposed to SARS-CoV-2 and possibly asymptomatic. Teachers had significantly higher antibody seropositivity than learners or parents. This may reflect higher vaccination rates, slower weaning of antibody response post exposure, or increased exposure risk. If due to increased risk, this supports the decision to prioritize teachers in the initial COVID-19 vaccination programs.

## Data Availability

Data availability will need to be requested from the corresponding author. The request will be considered with a concept note and reason for data access will be required. Data will only be shared after the minimum papers have been written by the Co-Principal investigators. Data will be made publicly available on the South African Medical Research Council (SAMRC) website after all the main papers have been published.
